# The Impact of the Adipose Organ Plasticity on Inflammation and Cancer Progression

**DOI:** 10.3390/cells8070662

**Published:** 2019-06-30

**Authors:** Luís Henrique Corrêa, Gabriella Simões Heyn, Kelly Grace Magalhaes

**Affiliations:** Laboratory of Immunology and Inflammation, Department of Cell Biology, University of Brasilia, Brasilia 70910-900, Brazil

**Keywords:** brown adipose tissue, white adipose tissue, pink adipose tissue, browning, cancer

## Abstract

Obesity is characterized by chronic and low-grade systemic inflammation, an increase of adipose tissue, hypertrophy, and hyperplasia of adipocytes. Adipose tissues can be classified into white, brown, beige and pink adipose tissues, which display different regulatory, morphological and functional characteristics of their adipocyte and immune cells. Brown and white adipocytes can play a key role not only in the control of energy homeostasis, or through the balance between energy storage and expenditure, but also by the modulation of immune and inflammatory responses. Therefore, brown and white adipocytes can orchestrate important immunological crosstalk that may deeply impact the tumor microenvironment and be crucial for cancer establishment and progression. Recent works have indicated that white adipose tissues can undergo a process called browning, in which an inducible brown adipocyte develops. In this review, we depict the mechanisms involved in the differential role of brown, white and pink adipocytes, highlighting their structural, morphological, regulatory and functional characteristics and correlation with cancer predisposition, establishment, and progression. We also discuss the impact of the increased adiposity in the inflammatory and immunological modulation. Moreover, we focused on the plasticity of adipocytes, describing the molecules produced and secreted by those cells, the modulation of the signaling pathways involved in the browning phenomena of white adipose tissue and its impact on inflammation and cancer.

## 1. Introduction

There is a strong correlation of cancer and obesity [1,2,3]. Considering the widespread occurrence of obesity and associated diseases, such as several types of cancer [4], numerous efforts are ongoing to lower the body weight gain through modulating the energy intake and/or expenditure [5]. It has been demonstrated that intentional weight loss can be an important tool to prevent cancer incidence [6,7,8,9].

**Figure 1:**
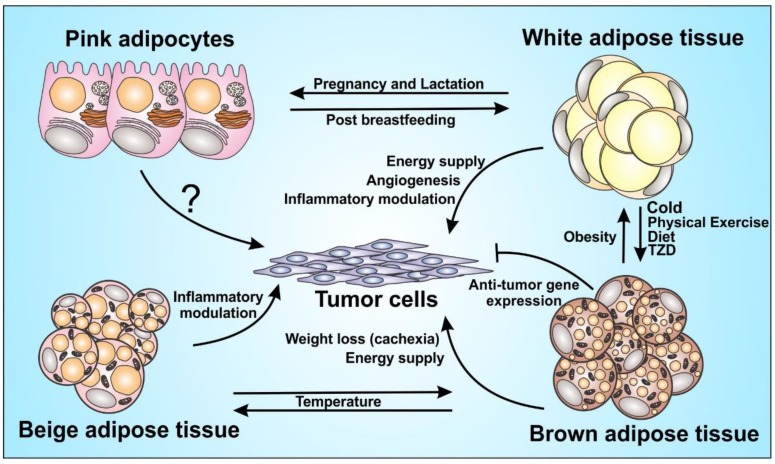
Adipocytes function in tumor progression. Adipocytes play a major role in the inflammatory modulation and endocrine function. It acts directly and/or indirectly on tumor progression, although some mechanisms are still unknown, such as the role of pink adipocytes on the tumor cells. The figure shows the great plasticity between brown, beige and white adipocytes with the influence of some inducers and the capacity of those cells to orchestrate the tumor microenvironment, through energy supply and immunomodulation.

Obesity is characterized by a chronic low-grade inflammation, and during the weight loss-process several changes in adipose organ can occur and modify the tissue’s composition and function, also remodeling of the immune cell landscape [10]. Weight loss can increase the presence of brown-like adipocytes in subcutaneous white adipose tissue, a process also known as browning or beiging [11], and this may have a significant impact in tumor microenvironment [12]. White adipose tissue from individuals with obesity can secrete a variety of inflammatory molecules that can strongly fuel cancer. Whereas brown adipose tissue can a therapeutic potential against cancer [13]. Cancer cell biology is directly affected by multiple cellular players present in the adipose tissue microenvironment. Therefore, cancer establishment and progression may be differentially influenced the different types of adipocytes, which have diverse morphologies and opposite functional roles (Figure 1). Since there are numerous complex biological processes underlying adipose organ and obesity, a detailed and accurate understanding about how obesity affects tumor biology at the molecular and cellular levels, can be useful to define the driving forces behind the obesity–cancer relationship.

Here, we will discuss the cellular and molecular pathways involved in the adipose organ plasticity and their different impact in cancer progression.

## 2. The Adipose Organ

The adipose organ consists of several subcutaneous and visceral depots [14] of adipose tissues with metabolic, regulatory, and plastic roles [15]. This adipose organ is mainly composed by adipocytes and several other cells and components such as lymphocytes, macrophages, fibroblasts, endothelial cells, and extracellular matrix. In the areas where this organ is mainly composed by white adipocytes it is called white adipose tissue (WAT) and where brown adipocytes are predominant it is called brown adipose tissue (BAT).

White adipocytes are characterized by its ability to store and release energy in form of lipids as a large and unilocular lipid droplet in the cytoplasm. WAT is the most abundant tissue in the body, and is not only related to the energy source of the body, but also acts as an important endocrine organ [16]. It is an active metabolic organ secreting several adipokines, such as leptin and adiponectin[17].

Brown adipocytes are characterized by the presence of high content of mitochondria and several small lipid droplets in the cytoplasm. BAT plays an important role in uncoupled respiration, via the uncoupling proteins (UCPs) present in their mitochondria. BAT is also a central location in the body for heat production (thermogenesis) [18] and it is deeply related to weight loss promotion [19]. It was initially believed that brown adipose tissue was found only in small mammals, hibernating animals and newborns. However, recent research has shown functional BAT present in adults at limited sites, such as the interscapular region and neck [20].

The adipose organ has prominent plasticity ability. White adipocytes can differentiate into brown-like adipocytes in WAT [21] in a process called beiging. Beige adipocytes are characterized by their multilocular lipid droplet morphology, high number of mitochondria and the expression of brown adipocytes genes (UCP-1, CIDEA, PGC1-α) [18]. Brown adipocytes raised in WAT are also identified as brite. These brite (brown-in-white) adipocytes are also known as beige, inducible brown or brown-like adipocytes [21]. Brite adipocytes can express UCP-1 and are originated in WAT in response to various stimuli. For instance, large unilocular white adipocytes can differentiate into beige adipocytes in response to cold or β3-adrenergic agonists [22]. However, these differentiated beige adipocytes can lose UCP-1 expression after mice are moved back to warmer conditions, demonstrating that the thermogenic profile of beige adipocytes is reversible [23].

**Figure 2:**
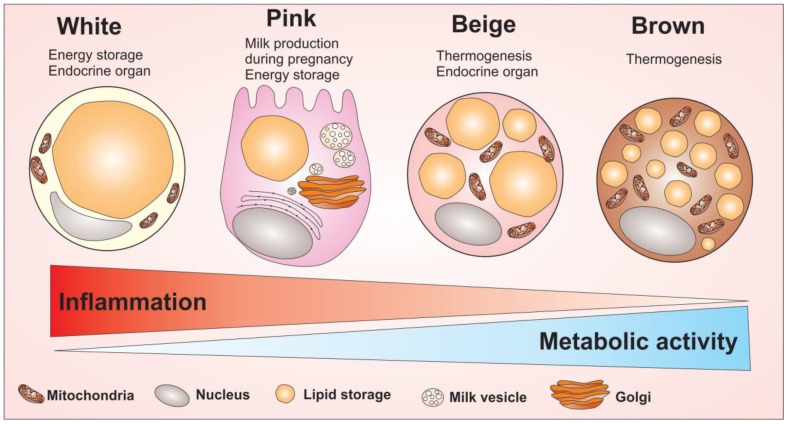
Adipocytes cells and their main morphological and cellular activity differences. Adipocytes are divided into four cell types that have different morphological and functional characteristics: the white, pink, beige and brown adipocytes. The white adipocyte is described as a cell with great energy storage ability, high pro-inflammatory profile, capable of producing several adipokines related to inflammation modulation. The pink adipocyte is described as a cell with a great potential for energy storage as well. However, this cell has a higher metabolic activity compared to white adipocytes, but low ability to regulate inflammation, compared to white adipocytes. Unlike white adipocytes, the brown adipocyte acts to maintain body temperature due to its high amount of mitochondria enriched with UCP1 protein. Brown adipocytes have small fat droplets spread throughout the cell and have a high metabolic activity. Between the two extremes of the white adipocyte to the brown, there is the beige adipocyte. The beige adipocyte has the ability to produce heat due to the high number of mitochondria, with the highest metabolic activity among all four types of adipocytes. Overall, the figure shows the morphological difference between the adipocytes cells as well as indicate the capacity to modulate inflammation and the metabolic activity of adipocytes.

A forth type of adipocytes has been described; the pink adipocytes [24,25]. Pink adipocytes are milk-secreting alveolar cells that can arise from transdifferentiation of white adipocytes during pregnancy and lactation. These cells are characterized by abundant cytoplasmic lipid droplets, apical surface with microvilli, roundish and large nucleus centrally located, a robust rough endoplasmatic reticulum (RER), Golgi complex, and milk-containing granules. Since the adipose organ acquires a pink color during pregnancy and lactation, these epithelial glandular cells were named pink adipocytes [24]. Evidence has supported the hypothesis of white-to-pink transdifferentiation, pink-to-brown transdifferentiation, and reversible brown-to-myoepithelial cell conversion [25], demonstrating once again the intense plasticity characteristic of adipose organ. The morphological differences among white, brown, beige and pink adipocytes are summarized in Figure 2.

Macrophages play an important role in adipose organ regarding the inflammation establishment. As adipocytes, macrophages can change their phenotypes dependent on stimulus. Adipocytes can recruit macrophages and polarize them to classical macrophage profile (M1) or alternative macrophage profile (M2) depending on the adipocyte status of inflammation [26]. In this context, M1 macrophages, characterized as pro-inflammatory, can induce Th1 response and strong microbicidal and anti-tumoral activity. The M1 or classical macrophage are sources of pro-inflammatory cytokines such as TNF-α, IL-1, IL-6, IL-12 and; and chemokines such as CXCL1–3, CXCL-5, and CXCL8–10. On the other hand, M2 or alternative macrophage can promote Th2 response, tissue repair, Treg recruitment, and tumor survival [27]. The levels of anti-inflammatory cytokines such as IL-10, IL-4 and TGF-β are elevated in this phenotype, promoting a favorable microenvironment for tumor development. In the context of tumor progression, the crosstalk between obese adipose tissue, macrophage polarization, and tumor cells is crucial for struggle tumor progression [28].

## 3. Brown Adipocytes and the Browning Process

The increase of brown adipocytes in adipose organ promotes an anti-inflammatory phenotype characterizing a healthy tissue development, decreasing insulin resistance, increasing thermogenesis, and consequently reducing obesity. The adipose tissue, when exposed to certain stimuli, such as cold, microbiota modifications [29] or adrenergic receptors (ADRB3) activation may undergo morphological and functional changes, in which white adipose tissue acquires characteristics of brown adipose tissue. This process is called browning [30]. The browning process has been highly studied due to its great anti-obesity and anti-tumor pharmaceutical potential. Recent studies have sought to target receptors in white adipocytes capable to induce the browning process in these cells, in order to reduce excessive fat accumulation, increase heat production and struggle metabolic diseases related to obesity.

Brown adipose tissue plays a major role in the hormonal regulation through the production and secretion of several adipokines, as well as the production of heat when exposed to cold. These characteristics were acquired throughout the evolution, turning possible the remodeling of white adipose tissue to beige/brown [30]. As previously described, BAT is characterized by a high number of mitochondria. In those organelles, located on the inner membrane, the amount of uncoupling protein 1 (UCP1) is enriched and its large number gives the brown color of the adipocytes [18].

The UCP1 protein is responsible for the production of heat in the brown adipocytes and its presence is increased in the browning process, being a great marker for the identification of the process [31]. Among the several factors capable to activate this protein in adipocytes and promoting thermogenesis, is Norepinephrine signaling through β3-Receptors [32]. Norepinephine can interact with three different types of receptors in brown adipose tissue. However, the interaction with β-adrenergic receptors is better described. The signaling cascade of norepinephrine is mediated via adenylyl cyclase activation, in which norepinephrine induces cAMP formation followed by a cascade of phosphorylation, including the activated protein kinase A [33]. Protein kinase A, in turn, can phosphorylate some transcription factors, including cAMP-response element binding protein (CREB) that will induce the transcription of some genes, including the UCP1 gene. UCP1 uncouples electron transport from ATP production, which in turn leads to controlled exothermic resolution of the electrochemical gradient and generation of heat to maintain body core temperature [19].

An important factor that may contribute to the remodeling of adipose tissue is the role of hormones, both paracrine and autocrine pathways. In addition to cold exposure, some hormones are produced in response to activate β3-adrenergic receptor and promote browning. Among the most studied hormones, we can mention catecholamines [34]. Peptide hormones such as fibroblast growth factor 21 (FGF21), are largely produced at lower temperatures and can bind to β3-adrenergic receptor, inducing the brown adipocyte phenotype in WAT. Studies have shown the important physiological role of FGF21 in the thermogenic process of WAT[35]. Mice deletion of FGF21 protein exhibits a large difficult adaptation when exposed to cold, resulting from the less efficient remodeling of white adipocytes to brown cells. FGF21 has the ability to act paracrine and autocrine in the adipocytes, to elevate the expression and activity of UCP1 in mitochondria. This regulation is due to the increase of adipose tissue PGC-1α protein levels [36]. Expression of UCP1 is controlled by some factors including PGC-1α. Thus, at low temperatures, the high production of FGF21 can induce the increase of UCP1 in adipocytes by increasing the expression of PGC-1α protein levels independently of mRNA expression.

In addition to FGF21, we can highlight the adipokine leptin, known for its function on hypothalamus receptors, controls energy expenditure and body weight. Leptin triggers browning of WAT through a network innervation of sympathetic nervous system fibers in BAT and WAT. Leptin promotes browning through peripheral and central mechanisms. Leptin-induced sympathetic activation increases energy expenditure in BAT, also leading to browning in WAT. Leptin also acts in skeletal muscle regulating of myogenic factors, such as the irisin, released after exercise, promoting browning [37]. The blockage of neural sympathetic fibers located in inguinal WAT prevents the development of brite adipocytes after cold exposure. The sympathetic tone is differentially regulated in adipose depots [38].

Thyroid hormones (TH) also orchestrate a key role in browning of adipose tissue. Studies have shown that THs act on the ventromedial nucleus of the hypothalamus (VMH) to inhibit AMP-activated protein kinase (AMPK), thereby regulating the browning process in adipose tissue, leading to increased thermogenesis and weight loss [39]. The participation of TH in this process has been experimentally proven. Studies have shown that hypothyroid mice had significantly decreased in interscapular BAT and hyperthyroid mice had significantly increased in this tissue activities, compared to euthyroid controls [40]. Furthermore, THs can interact with norepinephrine by controlling the process. Among factors that also may control UCP1 levels in adipose tissue is the concentration of triiodothyronine (T3) in adipocytes. T3 can be generated by the conversion of thyroxine (T4) to T3 by type II iodothyronine 5’-deiodinase (DIO2). In adipose tissue cells, DIO2 is activated through the sympathetic nervous system (SNS), in addition, adrenergic signals can be enhanced by THs which leads to an increase in thermogenic precession by the activation of UCP1 due to the increase in T3 concentrations [41].

In summary, several mediators are capable to change the adipocyte phenotype, inducing the production of some mediators that will act on the activation of UCP1 in the mitochondria of fat cells. Some of these factors are being extensively studied due the role of brown cells in the maintenance of tumor microenvironment, being a great target on the control of cancer progression.

## 4. Browning Process: Inductors and Mechanism

The multilocular distribution of lipids droplet, associated with the high amount of mitochondria, besides the expression of some characteristic BAT genes such as *Ucp1*, proliferator-activated receptor-gamma coactivator (*Pgc1α*) and *Cidea*, are some markers of the browning process [42]. The canonical process of browning in white adipose tissue begins with the sympathetic activation and norepinephrine release that will act by binding to β-3 adrenergic receptors in adipose tissue. After receptor activation, there is an increase in cAMP that will act by activating protein kinase A (PKA) [43]. Once activated, PKA will induce lipolysis by the phosphorylation of perilipin, which recovers the lipids by protecting them from the action of lipases on adipose tissue cells, releasing fatty acids (FA) [44]. This FA will be transported into mitochondria via carnitine palmitoyltransferase 1 (CPT1) which will then act in some ways to produce heat [45]. FAs can act both to regulate the expression of genes associated with thermogenesis, as well as to act regulating the activity of UCP1 proteins. After passing through the β-oxidation, FADH and NADH electron carriers will be generated, which will pass through the electron transport chain, generating a proton-motive force [46]. The protons pumped into the mitochondrial matrix through UCP1 generates energy that is released as heat [47]. When inside the mitochondria, the FAs also act modulating the activity of the UCP1s removing the purine inhibition, which generates an influx of H^+^ protons into the mitochondrial matrix, uncoupling oxidative phosphorylation and energy from the proton motive force dissipated as heat.

Cellular changes occur in adipose tissue to induce the thermogenic process. Several factors trigger the activation of β-3 adrenergic receptors increasing lipolysis, reducing the accumulation of FA concomitant with increased expression and activity of the UCP1 protein, leading WAT to a BAT profile.

The thermogenesis process of adipose tissue is orchestrated by several transcription factors and co-activators present in adipose tissue cells. These transcription factors act by regulating the genes responsible for production and activation of the proteins that play an important role in the production of heat and lipolysis of adipose tissues. Genetic transcription cascades have been widely studied and the mechanisms of action of some transcriptional regulators are already well defined, both for WAT and BAT [48]. Among the most studied regulators are mitochondrial protein transcription enhancers, as well as proteins related to the stability and formation of lipid corpuscles [49].

Peroxisome proliferator-activated receptor gamma (PPARγ) coactivator 1-alpha (PGC1α) complex is one of the main mechanisms of genetic regulation of the browning process [50]. Like most of the browning process regulators of adipose tissue, this complex is induced by external stimuli such as cold, exercise and fasting. The high concentration of PGC1α in adipose tissue is related to a higher production of UCP1 in the cells, as well as the expression of enzymes, are important for the respiratory chain in mitochondria [51]. Studies have shown that the specific tissue deletion of PGC1α followed by submission to cold leads to a decrease in the body temperature [52]. These results suggest that PGC1α exerts a role in the induction of browning, generating a physiological impact on the maintenance of corporal temperature. Although PGC1α is increased in WAT in the browning process, PGC1α is not required for differentiation of brown adipose tissue [53], however, PGC1α plays an important role in insulin sensitivity in adipocytes. In addition, studies suggest that PGC1α-deficient cells have an increased susceptibility to insulin resistance and type 2 diabetes in humans [54].

Regarding the importance of PGC1α in the remodeling of adipose tissue, there are several transcriptional regulators that control browning [55]. Other co-inducers of genes that are related to this process is PR (PRD1-BF1-RIZ1 homologous) -domain, containing 16 (Prdm16) [56]. In *ob*/*ob* mice model, Prdm16 is down-regulated. The leptin-deficient mice showed hyperphagia, impairment of insulin function, obesity and hypothermia. Prdm16 allows the activation of UCP-1 during BAT differentiation and specific genes related to browning [57]. The high fat diet-induced obese rats presented a downregulation of PRDM16 in a recent work which focused on physical activity and diets to modulate browning phenotype [58].

PRDM16 has been described as an important transcriptional regulator regulating browning in WAT [18]. Studies in mice have shown that an increase in the expression of PRDM16 is associated with the differentiation of WAT to beige adipose tissue in addition to the decrease of metabolic diseases. On the other hand, the deletion of this gene leads to a decrease in brown adipose tissue and an increase in some metabolic syndromes such as obesity [59].

As with PGC-1α, PRDM16 activity is also increased in cold exposure by acting on genes related to the production of mitochondrial-related proteins as well as in other gene regulators related to heat production [60]. Studies have shown that different depots of adipose tissue in the body of the organism have different abilities to undergo the browning process. Experimental data on murine models have shown that both epidydimal and visceral have less browning ability compared to subcutaneous WAT [61]. This different capacity of remodeling of the adipose tissue is due to the presence of regulatory genes in the adipocytes [62]. PRDM16 is one of these important genes that are found differentially in adipose tissues. PRDM16 can interact with WAT gene promoters by repressing its activity. Carboxy-terminal binding proteins 1 and 2 (CtBP1/2) are examples of genes reported as important promoters in WAT [63]. PRDM16 interact with these genes to inhibit the production of key proteins for the differentiation and functioning of WAT. PRDM16 significantly augments the amount of UCP1, CIDEA mRNA expression and FGF21 in epididymal WAT [62]. In addition, PRDM16 is required together with PGC-1α in the activation of PPARγ [64]. Both BAT and WAT require PPARγ for the differentiation and functionality of the adipocyte cells [65]. The post treatment with PPARγ agonist, rosiglitazone, shows an increase of UCP1 (main hallmark gene responsible for thermogenesis), which BAT and WAT participation is related.

The molecular mechanisms of browning control of adipose tissue have been the subject of studies for the development of pharmacological agents. Due to the key role of genes related to the biogenesis of mitochondria, as well as β-adrenergic receptors and inducers of UCP1 expression, agonists have appeared to induce WAT browning without the need for intensive exposure to cold and diets, through the molecular modulation of the process, aimed against obesity.

Due to the potential target of brown adipose tissue in the use of fat stock to produce heat, and consecutively to weight loss, ways of regulating the browning process of adipose tissue have been studied. The development of new browning inducers, as well as the use of thyroid target drugs to activate gene promoters has been described to boost WAT remodeling [66].

The practice of physical exercise modulate inflammatory factors in the body, including those that can act on the regulation of adipose tissue, increasing mitochondrial biogenesis [67]. This important regulatory ability turns physical exercise practice into a great contributor to the browning process. The practice of exercise may start browning by reducing inflammation as well as increasing pro-opiomelanocortin (POMC) neuron gene expression [68]. Initially, it was believed that POMC was a homogeneous population and responded similarly to hormones and nutrients, however, studies have shown its heterogenicity to responses to peripheral hormones, such as insulin and leptin responses [69]. Recent data have confirmed the performance of POMC in the browning process showing the synergistic performance of POMC, leptin and insulin. The practice of physical activities leads to a hypothalamic activation of the POMC population as well as a higher production of leptin and insulin that acted in the adipocytes, stimulating some fundamental genes for the remodeling of WAT, such as Ucp1, Cidea and Prdm16 [70]. In addition, periodic physical activity induces a greater production of FGF21 by muscle cells, which will act on the adipose tissues, promoting the greater expression of PGC1α [71].

Recently, several studies have shown the importance of the gut microbiota in the initiation of the browning process [29]. As well as the importance of physical exercises practice, the diet should also be controlled to reach a healthy microbiota. The connection of the gut microbiota interfering with the plasticity of adipose tissue became evident with the depletion of microbiota in mice, both with antibiotic treatment and in germ-free mice (GF) [72]. The absence of gut microbiota induced a significant increase in both inguinal subcutaneous and perigonadal visceral adipose tissue. The absence of the microbiota induces a high level of IL-4 and IL-13, as well as an increase in circulating eosinophils [73]. Both the presence of interleukin and eosinophils contribute to the attraction and polarization of macrophages to the M2 profile. Alternative activation of macrophages has been described as a contributing agent of the browning process in WAT, due to M2 polarization inducing the expression of tyrosine hydroxylase (TH) [74]. This enzyme plays an active role in synthesizing catecholamines on adipocytes, promoting lipolysis and consequently the production of heat through the mitochondria.

Furthermore, the gut microbiota triggers browning through the products of its metabolism. The gut microbiota-initiated trimethylamine (TMA)/flavin-containing monooxygenase 3(FMO3)/trimethylamine N-oxide (TMAO) pathway has been characterized as one of the major regulators of the process [75]. The flavin-containing monooxygenase 3 (FMO3) is a hepatic enzyme that, among its functions, acts on the metabolism of metabolites gut microbe-derived TMA to produce TMAO. As the end product of metabolism, circulating TMAO is indicative of cardiovascular disease [76]. In addition, high expression of FMO3 is also associated with obesity, and FMO3 knockdown or genetic deletion showed a higher positivity of UCP1 in WAT [77]. Although the role of the microbiota in the browning process is avid, little is known about its pathways of induction. However, several studies have been conducted to understand how products of bacteria metabolism induces browning of adipose tissue.

**Figure 3:**
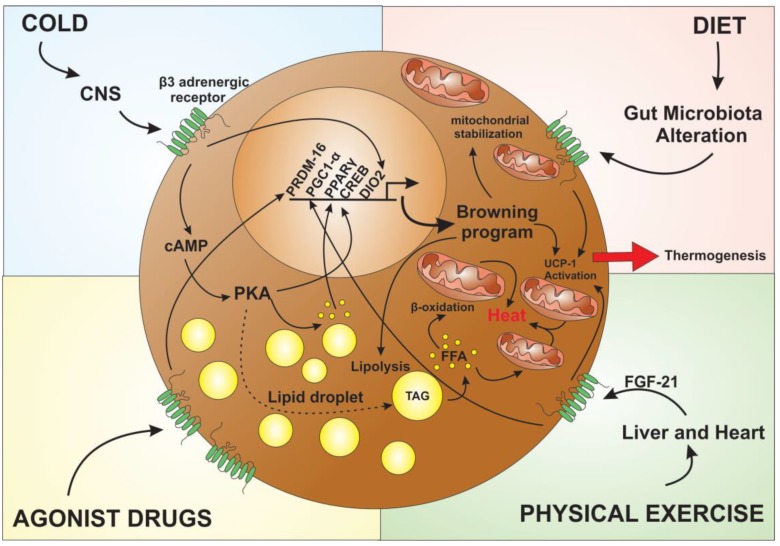
External agents capable of inducing the browning process in adipose tissue. β3 receptor activation regulate the transcription and activation of genes linked to mitochondrial activity and fat storage. The exposure to excessive cold induces release of catecholamines by the central nervous system (CNS), which will act on β3 adrenergic receptors on adipocytes initiating the signaling cascade. Activation of cAMP through the binding of catecholamines at adipocytes receptors will activate protein kinase A (PKA), which will then induce lipid droplet lipolysis. This lipolysis will induce thermogenesis by β oxidation in the mitochondria, as well as activation of the UCP1 protein, and the activation of important genes linked to the browning process like peroxisome proliferator-activated receptor γ (PPARγ), cAMP-response element binding protein (CREB) and Type II iodothyronine deiodinase (DIO2). Similarly to the cold, the food also influences the thermogenic process by the production of metabolites by the gut microbiota that will act both indirectly, acting in the liver, and directly in the adipocytes inducing the activation of the UCP1 protein in the mitochondria. The liver and heart act in the process being regulated by the practice of physical exercises increasing the release of fibroblast growth factor 21 (FGF-21), which will regulate the activity of the gene PGC1. In order to enhancer the process, several agonist drugs are also used, both in the increase of β3 adrenergic receptors expression and in the regulation of genes like PR (PRD1-BF1-RIZ1 homologous)–domain containing 16 PRDM16, acting in the regulation of expression and activation of UCP1, which will then potentiate the thermogenic process in the adipocytes, characterizing the browning process.

Further findings regarding the browning process induction pathways are making possible the use of pharmacological agonists that may activate the browning process. In addition, to the practice of physical activities, feeding and cold, hormonal regulators act at several key points in the body promoting greater expression of UCP1 (Figure 3). The main target of browning induction is through β3 adrenergic receptor [78]. CL 316,243 (disodium (*R,R*)-5-[2-[2-(3-chlorophenyl)-2-hydroxyethyl]amino] propyl]-1,3-benzodioxole2,2-dicarboxylate) is a potent l-adrenergic agonist [79]. Considering that β3-adrenergic receptor is significantly related to the onset of the browning process, the CL 316,243 (CL) has been studied as a potential anti-obesity treatment. CL-treated adipocytes have increased UCP1 expression, as well as treatment reduced lipid storage in these cells, thus showing that CL induced lipolysis in adipose tissue, followed by significant expression and activation of UCP1, leading to heat production.

Due to the fundamental role of PPARγ in adipogenesis, the use of PPARγ ligands as a cell differentiation control has become the target of several researches [80]. The role of PPARγ in lipid modulation is well established. PPARγ cooperates with other transcription factor families, including the C/EBPs and SREBPs, to regulate adipocyte differentiation. The role of PPARγ increasing BAT was shown by treatment with a potent PPARγ ligands such as rosiglitazone, an antidiabetic drug from thiazolidinedione pharmaceutical class (TZD) [64]. The treatment with this agonist leads to an increase in mitochondrial genes, such as UCP-1 and enzymes important for mitochondrial function such as cytochrome c oxidase (Cox), subunit VIIIb (Cox8b), and subunit VIIa1 (Cox7a1). Thiazolidinediones are important PPAR γ agonists leading to browning.[81]. TZD has been shown to be a major PPARγ activator in white adipose tissue, leading to the induction of PRDM16 complex with PGC-1α/β [82]. Although WAT already has a basal production of PGC-1α/β, studies have shown a large increase in the production of this protein, showing that increased activation of PPARγ via TZD can promote browning in WAT, via PRDM16.

## 5. Cancer and Adipose Organ

**Figure 4:**
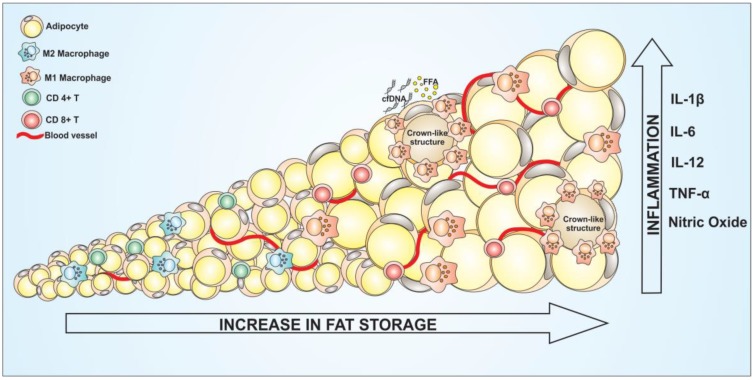
Increased lipid reserve in white adipocytes can favor inflammation. The imbalance in the energy reserve in fat cells leading to obesity is capable of mediating inflammation in tissue. Adipose tissue hypertrophy and hyperplasia can induce increase in vascularization, facilitating the recruitment of immune cells. Due to the secretion of various inflammatory mediators in a deregulated form in obese adipose tissues, there is an increasing polarization of immune cells to their pro inflammatory profile. The number of macrophages recruited into obese adipose tissue increases as well as its polarization to the M1 profile. Once polarized, macrophages are able to form crown-like structures (CLS) around adipocytes in the degeneration process, inducing their death with the release of cytoplasmic components into the extra cellular environment, this fact increases inflammation in the tissue, where more macrophages will be recruited and polarized. The release of cell-free DNA (cfDNA) by necrotic adipocytes increases the macrophages recruitment, which will then be polarized. In addition, the release of free fatty acids (FFA) may favor the development of some metabolic diseases such as cancer. Unlike healthy tissue, where a larger macrophage population is found in its M2 profile and CD4+ T cells maintaining tissue homeostasis, the obese tissue has an increased presence of CD8+ T cells, favoring the inflammatory process.

Among the many functions of adipose tissue, its main role is the energy storage. Especially the white adipose tissue, stores lipids in the form of triglycerides, to be used in the future as an energy source. However, imbalance in the storage of lipids from fat cells can lead to a metabolic dysfunction [83]. In view of the endocrine character of adipose tissue, a disorder may be associated with various metabolic diseases such as cancers, cardiovascular as well as liver tissues. As already described, adipose tissue is composed not only of adipocytes, but also of several other immune cells that act to maintain the tissue homeostasis [84]. The polarization profile of these immune cells in the tissue depends on the health status of the adipocytes. The type of inflammation predominant in the tissue can be important to define malignancy progress. Immunological cells can be recruited and polarized by external stimulus and modified to two different inflammatory profiles: type 1 and type 2 inflammatory phenotype [26]. The type 1 inflammation is described as a pro-inflammatory profile, and the immune cells polarized to this kind of inflammation is responsible for release pro-inflammatory cytokines such as TNF-α, IL-6, and IL-1β among another [85]. Deregulation on WAT leads to intense macrophage recruitment through chemokines release, followed by the polarization of these cells to M1 phenotype. The amount of CD8+ lymphocytes and natural killer are also increased in this tissue. On the other hand, the type 2 of inflammation is characterized by the presence of anti-inflammatory phenotypes, with the production and secretion of cytokines such as IL-10, IL-4, and TGF-β [86]. On the opposite way to type 1 inflammation, a higher amount of CD4+ lymphocytes and Treg cells can be found in the anti-inflammatory type 2 profile [87]. The interplay between adipocytes and immunological cells, such as macrophages and lymphocytes, may facilitate the establishment of several pathologies [88] (Figure 4). White adipose tissue from people with obesity display a crucial role in the increase of immunological cells infiltrate characterized by an intense presence of type 1 inflammation. Thus, deregulation of adipose tissue leading to obesity, can promote immunological polarization and adipokines and chemokines production. In addition, it can serve as energy source for tumor cells proliferation. A study showed that the gene expression profile from periprostatic adipose tissue of people with obesity and overweight patients is correlated with the risk for prostate cancer. The results showed changes in genes regulating adipogenesis, lipolysis, apoptosis and proliferation. It was observed that a downregulation of FADS1 (fatty acid desaturase) and an upregulation of LEP (gene encoding leptin), which is associated with adipogenesis, lipogenesis and ANGTP1 (Angiopoietin 1), is associated with vascular development. Periprostatic adipose tissues of obese or overweight patients presented altered expression of genes linked to adipose tissue activity, especially an increased inflammatory profile [89]. There is a crosstalk of adipose tissues and carcinomas through an increase of angiogenesis corroborated by higher expression of VEGF, proinflammatory cytokines such as IL-6 and TNF-α. The WAT, specially the visceral WAT, exerts a central role to develop a pro-tumoral secretome, associated to a higher risk for cancer in obesity [90].

The dysfunction of adipose tissue, due to obesity, associated with the tumor is characterized as a worse prognosis [91]. Due to the fact that obese adipose tissue is linked to an inflammatory condition, the modulation of the tumor microenvironment is influenced by the production of cytokines by adipocytes as well as immunological cells that are recruited and polarized at the site [92]. Obesity shows a deregulation of adipokines secretion, specially promoting a pro-inflammatory profile, as a result of higher adiposity and adipocytes dysfunction. In addition to the secretion of cytokines, adipokines are related to lipid metabolism, glucose and cardiovascular homeostasis. There is a group represented by acute-phase reactants, as C-reactive protein, plasminogen activator inhibitor 1, haptoglobin and amyloid A serum. The chemokines also exert a differential role, with higher secretion of MCP-1, CCL2, CCL5, MIP-2 (macrophage inflammatory protein-2). In addition to these factors, damage-associated molecular pattern (DAMP) or alarmins as HMGB1, heat shock proteins, tenascin C. Altogether, it can lead to an inflammatory microenvironment in obesity [93]

Cancers in general require a favorable environment for their progress as well as energy sources, as result of their high metabolic activity. Obese adipose tissue can provide these tumor growth and development factors through the large production of cytokines such as TNF-α, IL-6, IL-1β, and CCL2 [94]. These cytokines, among other functions, can establish an inflammatory environment by the recruitment of lymphocytes and macrophages. Once recruited into the tumor microenvironment (where there is interaction between adipocytes, matrix and tumor cells) by the deregulated adipocytes, macrophages tend to be polarized to their anti-inflammatory M2 profile by tumor cells [26]. Most tumors are characterized by high production of IL-4, IL-10 and IL-13. The association between the intense release of anti-inflammatory cytokines with the production of the chemokine leads to a substantial macrophage polarization on the tumor microenvironment. Once in the tumor site, the M2 macrophage profile is controlled by the tumor cells, in addition to an inhibition of the activity of anti-tumor immune cells, such as inhibition of TCD 8+ lymphocyte and NKT activation [95]. Moreover, adipocytes also play a role in this process. Studies have shown a significant reduction of macrophages in CCL2 knockout mice in the obesity-induced model [96]. This inflammatory profile is aggravated by the large infiltration of macrophages in WAT due to high levels of cell-free DNA (cfDNA). Obese adipose tissue in degeneration processes release DNA into the extracellular environment that leads to macrophage accumulation in the tissue [97]. Once in the tissue, macrophages can be polarized to the M1 profile leading to the formation of crown-like structures (CLS) [98]. Such structures are formed by the accumulation of macrophages M1 around adipocytes in degeneration. The macrophages then induce the death of these adipose, which leads to the release of cell components in the tissue, aggravating the inflammation. Furthermore, the cell death of these adipocytes will trigger the release of the fatty acids to the extracellular environment, which will be used as energy source by tumor cells [99]. Thus, obese white adipose tissue has an intimate relationship with the tumor, where there is the attraction of macrophages that will be, consecutively, polarized by the tumor as well as by the adipose tissue, aggravating the inflammatory state and favoring the tumor. Of note, adipose-derived stromal cells (ASCs) represent a crucial coadjuvant in the context of cancer development [100]. Desmoplasia, the extracellular matrix and stromal microenvironment remodeling process, impacts significantly on local inflammation, causing homeostasis disruption and facilitating the malignancy process [101]. On the tumor microenvironment, the extracellular matrix is stiffer than normal tissues. This happened in function of the high activity of myofibroblasts, the principal soldier in the desmoplasia process [12]. During the obesity, there is a significant increase in the myofibroblasts, which will acts depositing stiff matrix components such as fibronectin and fibrillar collagen, supporting the tumor establishment and inducing fibrosis on WAT [102]. ASCs, or just pre-adipocytes, have been described as a target to tumor progression because of the multipotent mesenchymal progenitor action. Tumor cells can release cytokines and chemokines such as IL-8 and growth-related α-protein (CXCL1), respectively. These cytokines and chemokines will recruit ASCs from WAT into the tumor microenvironment [103]. People with these two metabolic diseases, obesity and cancer, have been reported presenting a dysfunction on the IL-8 production, releasing higher amount of this cytokines by the tumor cells, resulting in an excessive ASCs recruitment to the tumor site. Once in the site, malignant cells induce the differentiation of ASCs into myofibroblasts via transforming growth factor-β (TGFβ)–mitogen-activated protein kinase (MAPK) signaling [104]. The presence of ASCs cells are related to a worse prognostic. Besides the ability to remold the extracellular matrix, the myofibroblasts can also give support to tumor cells by producing VEGF and inducing angiogenesis [104]. Moreover, cancer cells that remain following surgery can induce resident ASCs to promote tumor angiogenesis, exacerbating cancer growth and aggressiveness [105]. This is an important point to be considered, especially regarding the use of adipose tissue engraftment to build up the mammary glands following mastectomy surgeries.

Although the negative regulation that obese adipose tissue associated with the tumor may cause, the exacerbated secretion of leptin by adipose tissue form may be a crucial factor for cancer progression [106]. Leptin is mainly produced by adipocytes, although levels of leptin have already been detected by other tissues, such as skeletal tissue and placenta, but at lower levels. The main role of leptin is the regulation of appetite, acting on the central nervous system, reducing the appetite [107]. Leptin acts predominantly by binding to the full-length receptor OB-Rb, which will then induce the activation of the Janus kinase/signal transducer and activator of transcription (JAK/STAT) pathway, which will then induce the activity of phosphatidylinositol 3- kinase (PI3K) which promotes cellular growth, migration and invasion. In addition, the deregulated production of leptin by obese adipocytes leads to the production and secretion of inflammatory cytokines such as TNF-α and IL-6 by macrophage, modulating the polarization of lymphocytes, with the shifts the T-helper (TH) balance toward TH1 [108]. This action of leptin remodeling the tumor microenvironment has been increasingly studied, due to its great importance establishing low grade inflammation in obese individuals, increasing the risks of cancer.

Due to the great functionality of BAT and its high metabolic activity in the control of energetic homeostasis, this tissue has been studied for its ability to influence tumors development. This thermogenic capacity, the induction of lipolysis as well as the modulation of the inflammatory profile, are the main targets of the studies. Experimental analyses have shown a link between a super expression of anti-tumor genes and an increase of brown adipocytes cells in the adipose organ. One of the genes linked with browning and cancer is the PTEN gene. The PTEN gene acts on the regulation of cell growth and proliferation, being one of the most important anti-tumor regulators. This gene is considered a tumor suppressor for the action of its phosphatase protein product that is related with the regulation of cell cycle and the reduction of cell proliferation. With this in mind, a study demonstrated that overexpression of PTEN gene triggered resistance to cancer, energy expenditure and insulin resistance in mice [109]. In addition, the mice had high expression of UCP1, showing high BAT activity [109]. Although BAT has been reported as a healthy tissue, recent studies have shown its relation to cancer cachexia [110]. Cancer-associated cachexia (CAC) is a complex, multifactorial syndrome characterized by the loss of adipose and skeletal muscle tissues that leads to great weight loss, which is associated with a negative impact on patients’ survival. Studies in animal model of cachexia showed a great increase in the expression of genes associated with the browning process, such as UCP-1, Prdm16 and Pgc1α in patients with cancer [111]. Although this relationship with BAT promoting cancer-associated cachexia, suggesting adipose tissue dysfunction associated with a worse prognosis, further studies should be done to better elucidate the function of BAT in the tumor establishment.

The pink adipose tissue arises during lactation, changing the breast tissue microenvironment and promoting impact on the modulation of local immune cells [25]. Therefore, pink adipocytes could have a direct influence in breast cancer establishment. In breast tissue, white adipocytes can transdifferentiate into pink adipocytes, in a process called pinking, which is a reversible phenomenon. During this process, mammary epithelial secretory cells can lose the expression of PPARγ creating a pro-breast tumorigenic microenvironment [112], facilitating the establishment of breast cancer at this phase. However, the function of the pink fat cells on cancer progress, especially on breast cancer, need to be better characterized.

## 6. Conclusions

As a metabolic syndrome, people with obesity show a deregulation on the inflammatory profile. Changes of cytokines and adipokines secretion by adipocytes on the adipose organ may influence the immune system response, which is related to a worse prognostic on cancer development. In this context, the modulation of adipose organ has been studied, looking for a better treatment to use in association with a conventional treatment against cancer [113].

The white adipose tissue has been deeply implicated in cancer, whereas the increase of this tissue is highly related to a pro-tumorigenic microenvironment. Several pre-clinical, clinical and epidemiologic data [90] suggest that the increase of WAT characteristics is accompanied by a growth-promoting and pro-inflammatory microenvironment linked to increased cancer risk and/or progression.

BAT can work as a double-edged sword during obesity, since the induction of browning of the WAT can be a powerful tool to reduce the weight of people with obesity, facilitate weight loss and improve metabolic health, as we previously mentioned here. At the same time, the reduction of activation of the BAT can soften the cachexia status of an individual by inducing weight gain in muscle and increasing muscle mitochondrial biogenesis. Then, this last characteristic of BAT can be of great importance in cancer perspective. The inhibition of browning of the WAT can ameliorate the severity of cachexia that can occur in several types of cancer by increasing the energy expenditure [114]. Therefore, treatments that associate the reduction of inflammation with the blockade of β-adrenergic receptors could potentially ameliorate the severity of cachexia by reducing the browning of WAT. On the other hand, the activation of the brown adipose metabolism, as well as the induction of the browning of the WAT, can improve insulin resistance, reduce inflammation and increase the secretion of anti-inflammatory molecules, creating anti-tumorigenic microenvironment.

The plasticity of adipose organ components has been a target of the pharmaceutical industry and the medical research, aiming for the profile remodeling of the fat cells to a medicinal anti-cancer target. Therefore, the adipose organ is highly plastic, and this plasticity may be a useful pharmacological tool to combat cancer progression and support cancer therapeutic treatments.
